# Modeling genomic data with type attributes, balancing stability and maintainability

**DOI:** 10.1186/1471-2105-10-97

**Published:** 2009-03-27

**Authors:** Norbert Busch, Gero Wedemann

**Affiliations:** 1 System Engineering und Informationsmanagement, Fachhochschule Stralsund, Zur Schwedenschanze 15, 18435 Stralsund, Germany

## Abstract

**Background:**

Molecular biology (MB) is a dynamic research domain that benefits greatly from the use of modern software technology in preparing experiments, analyzing acquired data, and even performing "in-silico" analyses. As ever new findings change the face of this domain, software for MB has to be sufficiently flexible to accommodate these changes. At the same time, however, the efficient development of high-quality and interoperable software requires a stable model of concepts for the subject domain and their relations. The result of these two contradictory requirements is increased complexity in the development of MB software.

A common means to reduce complexity is to consider only a small part of the domain, instead of the domain as a whole. As a result, small, specialized programs develop their own domain understanding. They often use one of the numerous data formats or implement proprietary data models. This makes it difficult to incorporate the results of different programs, which is needed by many users in order to work with the software efficiently. The data conversions required to achieve interoperability involve more than just type conversion. Usually they also require complex data mappings and lead to a loss of information.

**Results:**

To address these problems, we have developed a flexible computer model for the MB domain that supports both changeability and interoperability. This model describes concepts of MB in a formal manner and provides a comprehensive view on it. In this model, we adapted the design pattern "Dynamic Object Model" by using meta data and association classes.

A small, highly abstract class model, named "operational model," defines the scope of the software system. An object model, named "knowledge model," describes concrete concepts of the MB domain. The structure of the knowledge model is described by a meta model. We proved our model to be stable, flexible, and useful by implementing a prototype of an MB software framework based on the proposed model.

**Conclusion:**

Stability and flexibility of the domain model is achieved by its separation into two model parts, the operational model and the knowledge model. These parts are connected by the meta model of the knowledge model to the whole domain model. This approach makes it possible to comply with the requirements of interoperability and flexibility in MB.

## Background

### Motivation

Molecular biology (MB) benefits greatly from the use of software technology in preparing experiments, analyzing data, and performing "in-silico" analyses. However interaction between MB software programs is often complicated.

This will be illustrated by an example from our work: Recently we localized short reads of nucleosomes, gained by Illumina Solexa technology, in a reference genome. The sequences acquired were analyzed by computing some of their properties and assigning annotations from the reference genome to them.

We used the programs RMAPQ [1], SOAP [2] and MAQ [3] for the localization. Each of them uses a separate input format, different from the short read data format. We had to perform three data transformations. Furthermore, the domain understanding of these programs is slightly different: SOAP and MAQ support pair-end sequencing, while RMAPQ does not; SOAP can report multiple hits, while RMAPQ and MAQ can not. Due to this different domain understanding, each program uses its own output format with slightly different semantic. We had to write three different scripts to obtain the nucleosome sequences from the reference genome. To relate the acquired locations with other annotations and visualize this data, we used GeneTrack [4], which is based on a more aggregated view of the acquired locations. GeneTrack does not evaluate single hits of the localization programs, it analyzes the number of hits on found locations (different short reads may point to the same location). GeneTrack uses proprietary data formats for both the acquired locations and the reference genome annotations. Again, we had to transform the output of RMAPQ, SOAP, and MAQ three times. And we converted the reference genome annotations from the gene bank format into the GeneTrack format.

This is only one example of limited interoperability. However, due to heterogeneous data standards and uncoordinated software development [5], there are many other scenarios of complicated data exchange. A shared domain understanding was identified as a potent means to achieve interoperability for MB software [5,6]. On the other hand, MB, especially the areas of functional genomics and proteomics, is a very dynamic research domain. The increasing number of biological databases [5,7-9] and the continuous development of the EMBL data format [10] attest to this fact. For example, the feature "snoRNA" was introduced with Release 69. In Release 92 the features "snoRNA," "scRNA," "snRNA," and "misc_RNA" were replaced by the new feature "ncRNA" and its mandatory qualifier "ncRNA_class." The qualifier defines the kind of "ncRNA," and its value set is defined in an extensible form [11]. This suggests that further development in the area of non-coding RNA may be expected in the future.

As new insights into MB are gained, the domain and its concepts quite often have to be viewed from a new perspective; sometimes new experimental or theoretical results shift even fundamental concepts. The term "concepts" means the notable entities in the domain, such as different kinds of molecules (DNA, RNA, and proteins) or special regions of molecules (genes or promoter). Software systems for MB must be sufficiently flexible to accommodate these changes in a reasonable amount of time; otherwise, the software soon becomes obsolete or its value diminishes [12].

Previous studies investigated extensibility as an important requirement to achieve flexibility for MB software; e.g. Jones and Paton [13]. Besides extensibility, changeability is equally required to achieve flexibility [14].

An approach often used to deal with the problem of flexibility is to consider only a small, well-defined area of the domain. By this means, knowledge changes are scarcer and, due to the lower program complexity, easier to implement. The drawback is an isolated domain understanding of these programs, which is very much dependent on the particular program's domain view. Furthermore, these programs very often use proprietary data formats. The explorative manner of biologists work is not well supported, and interoperability and data exchange are difficult to achieve. Performing large-scale "in-silico" analyses using several programs often requires an enormous amount of time and resources for interoperability. The challenge in developing software for MB is to satisfy both requirements appropriately: interoperability to support explorative work in MB, and flexibility to accommodate new knowledge in the domain.

To meet this challenge, we approach the problem from the fundamental level of computer program development; namely, the domain model, also known as "conceptual model." In object-oriented (OO) software development [15,16], the domain model is a systematic description of entities in the subject domain and their relationships [14,17]. It reduces the gap between mental and software models, and it supports communication between domain experts and software developers [18].

The domain model is created in an early development phase, the analysis phase. It is the basis for data models created in later phases, such as data formats for data exchange and file storage, or the database model, or the class model for the domain layer. Changes of the domain model affect many parts of a software system; they are therefore costly and complicated and should be avoided. So, besides flexibility, stability is also very important for domain models in MB. A comprehensive domain model for MB could provide a shared domain understanding for a wide range of programs and therefore support interoperability.

It is difficult to fulfill both requirements using commonly applied OO modeling approaches. In these approaches, domain concepts are modeled statically in class hierarchies with associations between classes. New or changed knowledge leads to changes in the domain model, which should be avoided, as mentioned above.

Therefore, we present a different modeling approach, which has adapted the "Dynamic Object Model" pattern [19]. This approach promises to fulfill both requirements of a comprehensive domain model for MB – flexibility and stability – in a balanced manner and therefore supports interoperability.

## Results

### Approach

#### Overview

The essential idea behind our approach is the separation of fundamental domain concepts from domain knowledge, as proposed by Beale [12]. Fundamental concepts define the scope of the subject domain, and hence the scope of the software. If fundamental concepts were to change, the subject of the domain and the software would have to change, too. Therefore, fundamental concepts constitute a stable core; changes would only affect domain knowledge.

Following this idea, fundamental concepts were modeled in an abstract class model and domain knowledge was reflected in an object model. This approach utilizes the "Dynamic Object Model" pattern [19], also known as "Adaptive Object Model" [20]. This is a composed pattern and its core is the "Type Object" pattern [21]. We named abstract classes corresponding to fundamental concepts *typed classes*. Instances of *typed classes *get a more concrete meaning from a special attribute, called *type attribute*, which describes a concrete concept. In other words, instances of *typed classes *are parameterized by their *type attributes*. These *type attributes *constitute the model for the domain knowledge. They are instances of special classes, called *type definition classes*.

**Figure 1:**
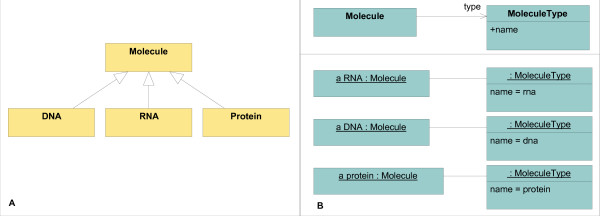
**Comparison of the classical and the proposed modeling approach**. A) Classical OO-class model of different kinds of molecules. B) Model of different kinds of molecules following the proposed approach. On top, the class model with *typed class *Molecule and its *type definition class *MoleculeType. At the bottom, the object model that defines concrete kinds of molecules, instances of MoleculeType. They are used by instances of the *typed class *Molecule.

The following example explains this approach: In classical OO models, different molecules such as DNA, RNA and proteins are represented as classes in a class hierarchy (Figure 1a). In contrast, the presented modeling approach uses classes as well as instances (Figure 1b): Molecule is a *typed class *and MoleculeType is a *type definition class*. Different molecule types are represented by instances of MoleculeType, not by subclasses of Molecule (see the object diagram in Figure 1b). These instances are *type attributes *of Molecule instances. By using this approach, new concepts can be introduced into the model without changing the class model.

**Figure 2:**
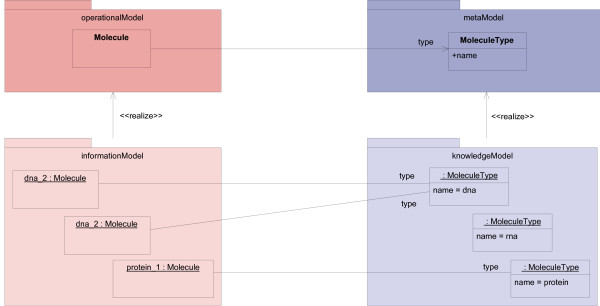
**Four different models**. The presented approach leads to four different models: operational model, meta model of the knowledge model (meta model), knowledge model, and information model. The operational model and meta model are OO-class models. The knowledge model consists of instantiated classes of the meta model. The information model consists of instantiated classes of the operational model that are parameterized by elements of the knowledge model. Elements of the operational and information model are shown in red. Elements of the meta model and knowledge model are depicted in blue. Instances are given in a lighter color.

Following this approach, we realized that four models can be distinguished on different abstraction levels (Figure 2):

• The **operational model **defines the scope of the domain model. It contains the fundamental abstract domain concepts. It is a classical OO-class model that consists of the *typed classes*.

• The **knowledge model **defines concrete concepts in the area of interest; these concepts and their relations are subject to changing knowledge. The elements of the knowledge model are instances of *type definition classes *that can be created and changed dynamically. They are used as *type attributes *in the operational model.

• The **meta model of the knowledge model **defines the structure of the knowledge model. It joins concrete concepts defined in the knowledge model with abstract concepts of the operational model. Classes in the meta model of the knowledge model are *type definition classes*. This model is therefore an OO-class model.

• The **information model **contains application data from the developed system. It is not developed in any phase of the software development process, but rather it originates during the execution of an implemented system. It consists of instances of classes from the operational model, which use type attributes.

The names "operational model" and "knowledge model" are inspired by the terms "operational level" and "knowledge level," used by Fowler in [22]. Beale used the terms "knowledge level" and "information level" in [12]; the latter term was the inspiration behind the naming of the "information model."

#### Properties of knowledge model concepts

**Figure 3:**
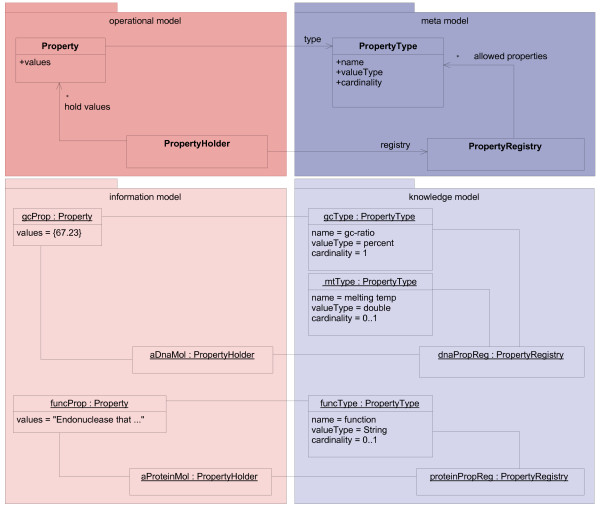
**Modeling properties**. Properties of concrete concepts are held by the *typed class *Property. Instances of its *type definition class *PropertyType (gcType, mtType, and funcType) specify the meaning of properties with their attribute name. The attributes valueType and cardinality restrict the data type and number of hold values. PropertyHolder and PropertyRegistry support the handling and definition of property sets. The PropertyHolder aDnaMol holds the Property gcProp of type gcType; the optional Property of type mtType is not set.

Concrete concepts usually define properties. For example, double-stranded DNA has a melting temperature and a GC ratio, and proteins can have annotations describing their function. In our approach, properties cannot be modeled as instance attributes of the *typed class*. This is because properties are not shared by all kinds of concepts; every concept has its own set of properties. To meet this challenge, we integrated properties using a generic property model (Figure 3). The basis of this model is the "Typed Dynamic Property" pattern [23].

The property model defines kinds of properties by instances of the *type definition class *PropertyType. These instances are elements of the knowledge model (the object diagram at the bottom right of Figure 3) and represent concrete kinds of properties (mtType, gcType, and funcType). Instances of the *typed class *Property (mtProp and funcProp) represent property values according to its PropertyType.

Domain concepts usually have several properties; they have a property set. These sets are defined by instances of the *type definition class *PropertyRegistry (dnaPropReg and proteinPropReg in Figure 3). These instances define property sets for concrete concepts, they are also elements of the knowledge model. The corresponding *typed class *for PropertyRegistry is PropertyHolder, which is a container class to manage instances of Property according to the PropertyRegistry.

The property model is integrated into our domain model using inheritance (see "Modeling properties of concrete concepts"). If inheritance might cause problems, delegation can be used instead: For example, when the class that should have properties is the subclass of another class, delegation would avoid problems that multiple inheritance could imply [24].

#### Associations between concrete concepts

**Figure 4:**
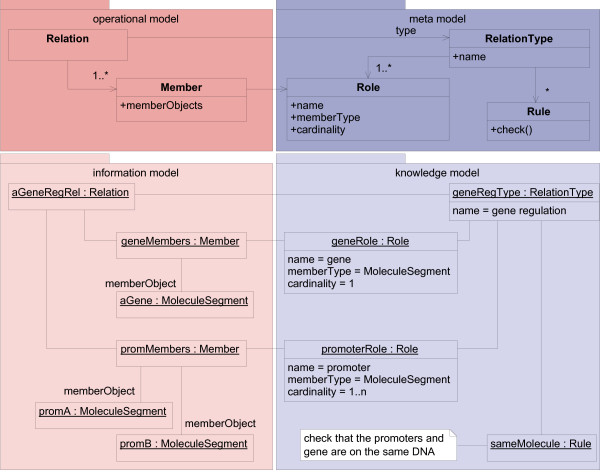
**Modeling relations**. A relation model realizes relations between concrete concepts defined in the knowledge model. Relation is a *typed association class *that holds references to its Members. The *type definition class *RelationType describes different relation kinds by its name attribute and defines Roles that members can play, and Rules that apply to the relation type. A Role can have more than one Member. The number of role members is restricted by the attribute cardinality. A Role furthermore restricts the type of its member objects by the attribute memberType (see memberType of promRole and associated memberObjects promA and promB). Rules related to a relation kind have to check that the relations are conforming to the rules (method check).

Domain objects are often related to other domain objects. For example, a promoter can be involved in gene regulation. To support relationships, Riehle suggests the "Relationship Type Objects" [19] pattern. It is a combination of "Association Classes" [22,25] (classes that describe associations) and the "Type Object" pattern. "Relationship Type Objects" are "Association Classes" that have a type attribute; in our terms, they are *typed association classes*. On the basis of "Relationship Type Objects," we have developed a model for associations between typed classes (Figure 4).

The main element of this model, the *typed association class *Relation, represents relationships between domain objects (Members). Its *type definition class *RelationType must be more complex than previously described *typed classes*. It defines Roles that can be played by Members, and Rules that apply to the Relation and its Members. The Role class is the *type definition class *of the Member class.

This is explained by an example (object diagram in Figure 4): In a relation, describing the gene regulation by promoters, the roles "promoter" and "gene" may exist.

Furthermore, the promoters and regulated genes must be on the same DNA-strand. aGenRegRel, an instance of Relation, realizes this relation. It is parameterized by the RelationType genRegType, which declares two Roles, geneRole and promoterRole. Members of the relation (aGene, promA, and promB) are managed by geneMembers and promMembers (instances of Member). Both are parameterized by its type attributes, geneRole and promoterRole (instances of Role). The Rule instance sameMolecule is associated with geneRegType to ensure that all members of a geneRegType relation are located on the same DNA-strand. The logic used to perform the necessary checks cannot be shown in the object diagram, so a UML note is used to declare the logic.

### Structure of the model

#### Modeling linear biological macromolecules

**Figure 5:**
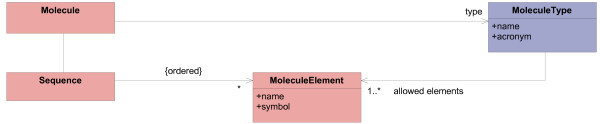
**Model of molecules**. This model part describes linear biological macromolecules. Molecule, Sequence, and MoleculeElement are part of the operational model. MoleculeType enables the definition of different molecule kinds.

DNA, RNA, and proteins are the main domain concepts. They are polymers, consisting of nucleotides or amino acids. It is common to present these molecules as a sequence of their elements. Figure 5 shows the model of linear biological macromolecules.

Many different kinds of molecules exist, for example ordinary DNA or RNA and more specific cDNA, tRNA, and various ncRNAs. The relevance of molecule kinds depends on the application; sometimes a less relevant kind may become important over the course of research. In order to support changeability, molecules are modeled as the typed class Molecule and its *type definition class *MoleculeType.

The linear structure is modeled by Sequence and MoleculeElement; Sequence reflects the order of elements. The association between MoleculeType and MoleculeElement is used by instances of MoleculeType (they are elements of the knowledge model) to define the set of MoleculeElements, which can be contained in a specific molecule type.

This model makes the dynamic definition of molecule types possible; new types of molecules can be defined without creating a new class.

#### Modeling special regions in linear macromolecules

**Figure 6:**
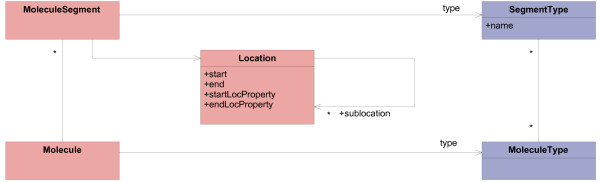
**Modeling special regions of molecules**. This model part describes special regions within linear macromolecules. MoleculeSegments are located at a certain Location in Molecules. This is part of the operational model. SegmentType is part of the meta model. It enables definitions of special regions in the knowledge model.

Molecules can have special regions in their sequence, such as genes and promoters in DNA or DNA-binding regions in proteins. Figure 6 shows the model of special regions and its relation to the model of molecules.

An important attribute of a special region is its location in the sequence. Simple locations only have a start and an end position. However, there could also be more complex locations:

I. They can have gaps; e.g., the areas of introns are not part of coding sequences.

II. They can be located between two molecule elements; e.g., the cleavage site of a restriction enzyme.

III. The start or end position could only approximately be known.

For these reasons, the operational model contains the class Location. Its attributes startLocProperty and endLocProperty specify the meaning of the start and end values. They allow the definition of special positions corresponding to (II) and (III). The self-referencing association sublocation can compose locations to deal with gaps (I). If a composed location contains sublocations which are not adjoining, it contains a gap.

The class MoleculeSegment represents special regions within molecules. However, there is a wide variety of special regions, and it is expected to discover new region types in the future (see the example of "ncRNA" feature in the EMBL format in "Motivation" section). Therefore, MoleculeSegment is a typed class with the *type definition class *SegmentType. This allows the integration of new kinds of special regions into the model, e.g., when new kinds of ncRNA become interesting. Which special regions can occur in a molecule depends on the molecule type. Therefore, an association between MoleculeType and SegmentType describes allowed segment types.

#### Modeling properties of concrete concepts

Simple properties as described in "Properties of knowledge model concepts" are not sufficient in MB. For many properties their values and equally further information about the values is important. We named this information "annotations." An example of such a property is the melting temperature (TM) of a doublestranded DNA, calculated from the nucleotide sequence. Different TM-algorithms exist and many factors affect their suitability to calculate TM. In order to assess a TM value, it is important to know how the value was calculated.

**Figure 7:**
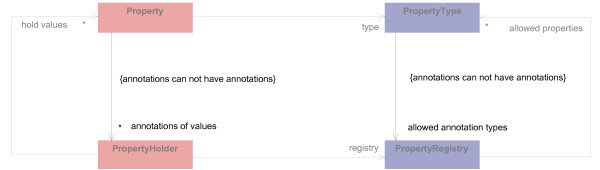
**Extension of the property model by annotations**. Values of properties of concepts defined in the knowledge model can have annotations, represented by the association annotations of values. This association has a constraint by which a Property, that is itself an annotation, cannot have annotations. The annotations that a Property can have are restricted by the association allowed annotation types. Classes and relations previously explained are shown in gray.

These special characteristics of properties are considered in an extended property model (Figure 7). Gray parts are taken from the simpler model above; black parts are new to the model. The new association between Property and PropertyHolder enables Property to hold annotations of its values. The one-to-many cardinality allows each property value to have annotations and its individual PropertyHolder. A constraint on the association prohibits annotations from having further annotations. Annotations of an annotation would not be of value. Their responsibility is to clarify the property value meaning. Annotations must be so precise that an explanation of them is not needed. PropertyType uses an association with PropertyRegistry to define which annotations are allowed for its values. To avoid the specification of an annotation set for annotations, a constraint prohibits the definition of a PropertyRegistry for annotations.

**Figure 8:**
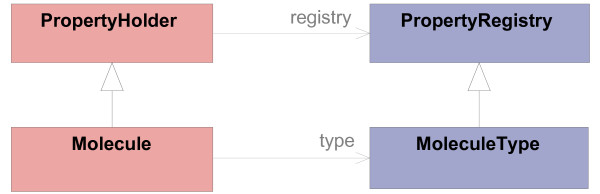
**Example of the usage of properties**. The property framework is integrated into the operational and meta model by inheritance. This is exemplified here by the Molecule class.

Typed classes of the operational model (Molecule, MoleculeSegment and Relation) inherit from PropertyHolder to enable them to hold properties. Therefore, the corresponding *type definition classes *have to be subclasses of PropertyRegistry. This is illustrated by an example for the classes Molecule and MoleculeType in Figure 8.

#### Overview of the operational model

**Figure 9:**
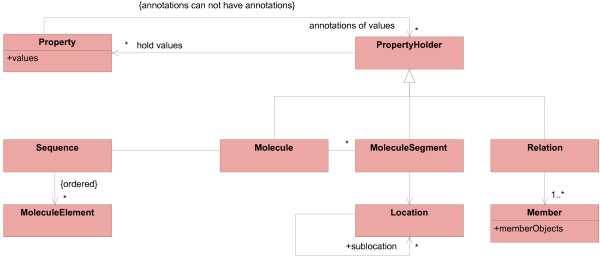
**Operational model**.

The preceding sections explained parts of the operational and the meta model in combination. The next two sections will explain both models separately to clarify their content and responsibility. This section describes the operational model (Figure 9).

Molecules and special regions within these molecules are the relevant concepts in the domain. They define the scope and have to be contained in the operational model. Both are represented by *typed classes*, Molecule and MoleculeSegment. Relations between molecules and special regions are also in the focus. They are realized by the relation model described in "Associations between concrete concepts." The operational model contains the *typed class *Relation for this reason.

Instances of these typed classes have to hold type specific properties. Therefore, Molecule, MoleculeSegment, and Relation are subclasses of PropertyHolder.

The linear molecule structure is represented by the class Sequence, while the class MoleculeElement represents the monomers. The class Location specifies the position of a special region within a molecule. Member holds references to objects involved in associations; these objects are usually Molecules or MoleculeSegments.

The operational model defines linear biological macromolecules, special regions within these molecules, and relations between them as concepts in the scope of the domain model.

#### Overview of the meta model

**Figure 10:**
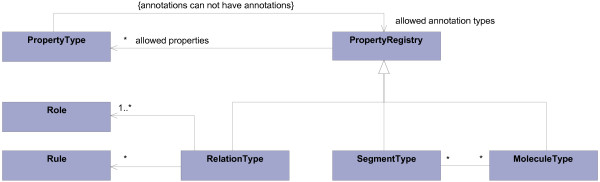
**Meta model of the knowledge model**.

Figure 10 shows the meta model for the knowledge model. This model defines the structure of the knowledge model and basically consists of the *type definition classes *MoleculeType, SegmentType, and RelationType. They allow the definition of new kinds of molecules, molecule segments, and relations by creating instances of them. The definition of property sets is supported by subclassing PropertyRegistry.

The *type definition class *RelationType supports the definition of member roles and the assignment of relation type specific rules by the associations to the Role and Rule classes.

The meta model of the knowledge model provides means to introduce new domain concepts, including their relations and properties, without needing to define new classes.

## Discussion

### Improvements due to the modeling approach

This work presents a domain model for MB, based on a novel modeling approach. In contrast to traditional modeling, this approach provides means to develop a single comprehensive view of the MB domain. Therefore, it supports a higher degree of interoperability (see "Effects on interoperability").

The strength of the modeling approach is the separation of the model into operational and knowledge models. The operational model defines the scope of the model at a high abstraction level, (see "Overview" and "Overview of the operational model"). This ensures that all relevant concepts can be represented in the model, albeit on a very abstract level.

The knowledge model decreases the abstraction level. It describes concrete domain concepts using objects that are applied as type attributes of classes in the operational model. New or changed domain concepts can be flexibly defined in the knowledge model (see "Overview" and "Overview of the meta model.")

The separation of the knowledge model and usage of instances facilitate the utilization of semantic technologies, e.g., ontologies (see "Use of ontologies as the knowledge model"). These technologies are more suitable for knowledge representation than commonly used class diagrams; they enhance the clarity and strength of the model.

An increased changeability, necessary due to the knowledge development in MB (see "Motivation"), is also supported by this approach. The applied design pattern "Dynamic Object Model" is intended to support changeability better than traditional approaches. Furthermore, the "Effects of changing knowledge" section demonstrates the limited effects of changing knowledge on implementations that utilize the presented modeling approach. These limited effects make changes easier and less time consuming. New findings can be reflected faster and with less effort in the software. Furthermore, changes arising from new knowledge only affect certain parts; hence there is less chance of introducing errors into the software. Positive effects in terms of maintainability and software quality are discussed in "Consequences for software development."

### Consequences for design and software development

Due to the fundamental role of domain models, the manner in which they are developed strongly affects the design of software and the development process. Consequences of the modeling approach for software design concern the internal software structure (classes or components) as well as the interaction of software components. In this article, the term "component" denotes a small modular and replaceable part of the system that provides a determined functionality [25,26].

#### Consequences for software development

The modeling approach distinguishes the abstraction level by using different models: the operational model, the knowledge model, and the meta model of the knowledge model. These models use the same modeling technology. At first glance, systems based on this approach seem to be harder to maintain than traditionally developed systems. Developers have to put significant effort into understanding this kind of modeling. However, we and others have observed that developers who manage to achieve this understanding find such systems easier to maintain than traditionally developed systems [27].

Furthermore, the separation of operational and knowledge model reduces coupling between concepts on different abstraction levels. The operational model is notably smaller and less complex than the class model of traditionally developed systems. Both of these factors – a good separation of concerns and lower complexity – are known to support maintainability and enhance software quality [14].

#### Consequences for the structure of software components

Systems based on this approach need typical components in order to handle the knowledge model, usually a repository that provides a list of known types. For example, for the instantiation of typed classes, a value for their type attribute is needed. This value can be obtained from the repository.

Another typical component is a persistence component that loads the knowledge model from permanent memory. Standard methods for mapping object models like the knowledge model into relational databases and XML files can be used in this persistence component [28]. The viability of this approach is shown by the implementation of the MB framework "Molecule Computation Kit" (MCK), which is based on the domain model presented here (see "Validation of the domain model" below). The current MCK version uses XML files to define the knowledge model. In the further development of MCK, however, ontologies seem to be a promising alternative for defining the knowledge model, as discussed in "Use of ontologies as the knowledge model."

In the presented modeling approach, the formal description by *type attributes *is advantageous for the implementation of software components. Thanks to this description, components can handle data generically and hence they are unaffected by changing knowledge. For example, MCK uses generic writers and readers that store and load molecules, molecule segments, and relations between molecules by utilizing *type attributes*. The MCK also contains a design study for user interface components that uses the formal description to display properties of molecules.

However, generic handling is not always possible. For instance, new domain concepts are usually followed up by new data processing components that process these concepts. These components must be integrated into the system. For this reason, a software system has to provide extension points for the integration of new data processing components.

As another example of the advantages of the modeling approach presented here, data processing components can use type attributes to validate input data with regard to the concrete type of a *typed class *or necessary properties. This generic validation even allows the processing of concepts that were unknown at the development time of the data processing component; an inherent interoperability is achieved.

Even data processing components themselves, including their I/O ports and configuration parameters, could be described by type attributes. This would allow the dynamic and semantically correct composition of data processing components [29,30] and support interoperability and the explorative manner of biological research. This interesting approach is the subject of further investigations (see "Integration of data processing components").

#### Consequences for the interaction of software components

The use of *typed classes *also concerns the interaction of system components. During interaction, the involved components must act in accordance with the meaning of the exchanged data. This requires the ability of components to utilize the *type attribute*; since the meaning of instances of *typed classes *depends on the value of their *type attribute *and their class. As described in "Consequences for the structure of software components" generic components must utilize the *type attribute*. They depend on software structures that allow them to read the meta data provided by the *type attributes*. A modular design of these software structures allows their reuse by non-generically implemented components. The *type attributes *can then be read and utilized by all components in internal communication.

Since changes in the knowledge model may often occur, data using different knowledge model versions will exist. This becomes relevant for reading older data from files or importing data from system installations with other knowledge model versions. Transformation rules support persistence components in this task. They describe steps necessary to convert data from one version to another, and would support automatic data transformation.

The modeling approach presented here supports the development of transformation rules. Hence the separation of the knowledge model makes changes to the domain understanding explicit. A change in the knowledge model indicates the necessity for developing a transformation rule. Furthermore, the reasons for the knowledge model change are known. This knowledge is helpful in developing the transformation rules; due to the explicit presence of the change, this knowledge can be used immediately.

### Effects of changing knowledge

One of our aims was the development of a domain model for MB that allows the fast and convenient integration of new knowledge while providing a stable basis for software development. To assess the achievement of this goal, we have analyzed the effects of changing knowledge on implementations based on the presented approach.

When new domain concepts arise, or when they are changed, the class model of the domain layer is not affected. In the case of changing domain concepts, it is sufficient to create new instances of *type definition class*es, or to change existing instances. In traditionally developed systems, a new class must be created as mentioned in "Motivation," significantly more time and effort is needed than in the presented approach. Hence, in contrast to traditionally developed systems, our approach separates the software implementation from the knowledge representation, so changes in knowledge have limited effects on the software implementation.

Changing knowledge only affects components directly dependent on them, so they must be adapted. Generically implemented components and components not involved remain unchanged. For example, the changed cardinality of a concrete concept property affects the implementation of processing components that must consider this property in its processing. Components that work with the same concept but do not use the changed property will not be affected. Our practical experience with MCK supports this statement

A close examination of possible changes reveals three different kinds of changes to the knowledge model: (i) The introduction of new domain concepts leads to new model elements. (ii) Domain concepts must be removed. (iii) Modified domain concepts lead to modified model elements. These different kinds of change must be handled in an appropriate manner.

We developed the following strategy to deal with the different kinds of changes and applied it to the MCK. New and modified knowledge model elements can mostly be handled generically. When this is not applicable, the strategy suggests ignoring unknown elements of the knowledge model. This is possible because a software system that is not aware of a model element will not depend on it.

Simply removing obsolete elements from the knowledge model would also lead to "unknown" elements. However, these removed elements must not be handled generically; they were discovered to be obsolete and should not occur in the system. To prevent generic handling of these elements, it is not sufficient to simply remove them. They have to remain in the model and must be flagged as obsolete.

### Effects on interoperability

The limited interoperability of current MB software motivated the development of our domain model. The consequences of our approach for interoperability are outlined in this section.

In "Motivation" we demonstrated that a shared domain model is a potent means of achieving interoperability. This does not mean that the interacting systems must use the same software implementation. It is sufficient that they use the same domain model. The presented modeling approach provides the flexibility and strength required to develop a widely used domain model for linear biological macromolecules. Systems using this domain model are able to exchange data simply and accurately, thus supporting interoperability on a new level.

The use of meta data in information systems is known to support interoperability [31]. A shared domain understanding is usually not given in communication between systems with different domain models. A mapping that transforms the data of one system into a form understandable by the other system is necessary. The development of this mapping is usually a complicated process and often associated with loss of information (see "Motivation"). And it is important that this mapping does not modify the semantics of exchanged data.

The section "Consequences for the structure of software components" describes how data processing components can use *type attributes *to validate their input data. This kind of validation signifies a shift from type-based communication to content-based communication. Type-based communication only considers data types specified by method signatures to ensure correct communication. Content-based communication considers not only the data type, but also the semantics of data by utilizing meta data like the *type attribute*. For example, content-based communication can distinguish between a float value describing a percent value and one describing a temperature value. The presented approach allows systems to communicate in type-based and content-based manner. This achieves greater flexibility in the mapping development and leads to better interoperability.

### Validation of the domain model

Our domain model must be able to include the relevant concepts of the domain. To validate this, we successfully proved that the data structures of the EMBL standard [10] can be mapped into our model, and developed an according mapping. EMBL database entries were handled as molecules and EMBL features were treated as molecule segments. Other EMBL line codes were mapped to properties and annotations. Qualifiers of EMBL features were handled mostly as annotations for segments (EMBL features). We verified this mapping by implementing a parser for EMBL files in our MCK, which will be described next.

We demonstrated the feasibility of systems based on our domain model with a framework for linear biological macromolecules. It is called Molecule Computation Kit (MCK) and is available at .

Generic models like our domain model do not provide type safety by themselves. However, type safety provides strong type checks at compilation time and many errors can be detected early.

In order to compensate for missing type safety, we use runtime type checks against the *type attributes *in the MCK. To make these runtime type checks transparent and decrease complexity, we implemented the property model in a set of service classes. They manage properties and perform type checks.

In order to guarantee that annotations do not have further annotations (see "Modeling properties of concrete concepts"), we distinguished two different kinds of properties and introduced corresponding classes. We named them Feature, which can be annotated, and Annotation, which can not have further annotations. All typed classes of the MCK use the service classes by inheritance.

Furthermore, we implemented an XML-based storage component. It is based on the structure of the operational model and handles all kinds of molecules, segments, and relations between molecules. It utilizes the formal description of concepts provided by the *type attributes*. Generic readers and writers are assigned to data types. Annotations and Features of stored objects are handled by these readers and writers according to the data type specified by the PropertyType. Therefore, the storage component does not have to be changed when new knowledge model elements are defined. Due to its generic implementation, the storage component can work with different knowledge model versions.

### Similar work

Our domain model uses solutions from the composite design pattern "Dynamic Object Model" [19,20], which has been successfully applied in different domains. The developed model meets the special requirements of MB by adapting the pattern. The *typed classes *and their *type attributes *are similar to the "Type Object" pattern [21]. The property model applies the "Typed Dynamic Property" pattern [23], and the model for associations uses ideas described as "Relationship Type Objects" in [19].

The main difference of our approach compared to "Dynamic Object Model" is the explicit distinction between operational and knowledge model and the explicit description of the knowledge model structures by the meta model of the knowledge model. The information processing model is strictly separated from the knowledge representation. This separation is also intended by the "Archetype" modeling approach of Beale [12] and the "Knowledge Level" described by Fowler [22]. Beale proposes the proprietary archetype definition language (ADL) to specify the knowledge model. The ADL has a higher expressiveness than the currently used knowledge model description. However, the ADL has not yet been broadly applied. In contrast, we use well-known OO technology, which we are going to combine with ontologies to derive the knowledge model from them (see "Use of ontologies as the knowledge model"). By this means, the knowledge model would get significantly greater expressiveness, and well-known technologies would be used.

Jones and Paton analyzed and described modeling constructs to achieve extensibility in data formats for functional genomics [13]. They described how different modeling constructs support frequently performed tasks, mainly in data analysis. Although they did not focus on domain models, some of the described constructs are suitable for domain modeling and were adapted in our approach.

Our property model extends the Name-Value-Type triples (NVT) described in [13] by a description of data types and cardinality. This extension supports the "reasonable" usage of NVTs as demanded in [13] and increases semantic expressiveness.

In [13], inheritance was also described as a means to achieve extensibility. However, we do not use inheritance for extension on the knowledge level. Inheritance is only used on a more technical level, e.g., to define extension points for generically implemented or data processing components. Inheritance is not used to represent "is-a" relations between domain concepts. This kind of knowledge representation is too inflexible for MB domain models (see section "Motivation"). Furthermore, inheritance often causes misunderstandings between domain experts and software engineers. Domain experts often think of "is-a" relations as restrictions, e.g., a cDNA is a DNA without introns. In contrast, commonly used OO programming languages and the UML interpret "is-a" relations described by inheritance as an extension by attributes, methods, or associations.

Ontologies provide a stronger way to describe relations between domain concepts. Not only "is-a" relations, but also "has-a" relations and even other relation types can be described by ontologies. Jones and Paton also described ontologies as a suitable means to achieve extensibility in [13]. We share this opinion, and will derive the knowledge model from ontologies in the future (see the next section).

### Use of ontologies as the knowledge model

#### Motivation for the use of ontologies

In MCK we currently identify concepts in the knowledge model by simple name attributes of the *type definition classes*. This does not allow the expression of semantic relations such as "is-a" relations between general and specialized concepts.

The usage of ontologies is promising to cope with this problem. Ontologies provide a formal description of domain concepts and of relations between them, as "is-a" and "has-a" relations. "Reasoners" (components of ontology software packages) can use this description to determine semantic associations like "a snoRNA is-a ncRNA." There is an active community for bio-ontologies [32-34], and ontologies have taken an important place in bioinformatics [33,35]. Their semantic capabilities were applied in several fields, such as data exchange, information integration, search and query of heterogeneous data sources, and computer reasoning [36].

An adoption of ontologies will help to acquire a representation of "is-a" relations between domain concepts [13,37]. We have developed a strategy to derive the knowledge model from ontologies. However, a full description of this strategy would be beyond the scope of this paper and will be the topic of an upcoming publication. Therefore, we provide a sketch of our strategy in the next section.

#### Strategy to derive the knowledge model from ontologies

The essential idea to derive the knowledge model from ontologies is to use the concept identifier of ontological concepts as the name attribute value of *type definition classes*. A mapping between ontological key concepts and *type definition classes *provides entry points into the ontology. "Is-a" relations in the ontology are used for navigation from entry points to ontological child concepts. So, one can recursively derive the descriptions of concrete concepts for the knowledge model. Properties of ontological concepts and "has-a" relations can be used to derive property sets of the concepts.

An example illustrates the strategy. It uses the Sequence Ontology, Version 2.3 [34,38] and applies the notation "term-ID = term name" to reference ontology terms: The term "SO:0000110 = sequence_feature" is identified as a key concept in the ontology and is mapped to the class SegmentType. This term serves as an entry point, and according to this term, an instance of SegmentType is created and inserted into the knowledge model. The concept id "SO:0000110" is used as the name attribute for the new instance.

All ontology terms with a direct or indirect "is-a" relation to the term "SO:0000110 = sequence_feature" are searched by reasoning. Every term contained in the result forces to insert a corresponding instance of SegmentType into the knowledge model. For example, the term "SO:0000001 = region" has a direct "is-a" relation to "SO:0000110 = sequence_feature" and is inserted into the knowledge model. The term "SO:0000833 = transcript_region" has an indirect "is-a" relation to "SO:0000110 = sequence_feature". This means, there is a path from "SO:0000110 = sequence_feature" over "SO:0000001 = region" to "SO:0000833 = transcript_region". Therefore, "SO:0000833 = transcript_region" is inserted as a SegmentType into the knowledge model.

This strategy permits the derivation of the knowledge model from ontologies, and "is-a" relations (but also other relations) can now be expressed in the knowledge model by using reasoning.

#### Current usage of ontologies and their relevance for deriving the knowledge model

Ontologies are used extensively in current MB software and our approach can benefit from experiences made there. This section provides a short overview of current ontology usage, relevant to our approach.

The TAMBIS project [39] is an example for ontology usage to mediate between different domain models. A mediator component and several database wrappers use the TAMBIS ontology to provide transparent access to heterogeneous databases. The TAMBIS strategy is to perform a mapping between the terms of the heterogeneous databases and the TAMBIS ontology terms. TAMBIS usage may be convenient for the user. However, due to continuous database development, the wrapper maintenance will be very labor intensive. On the other hand, the TAMBIS ontology proves the possibility of a comprehensive domain ontology.

Another approach is to integrate ontologies into data models, e.g., FuGE [40] and MAGE-OM [41]. In both models, classes, representing domain objects, use associations to the class OntologyTerm, that represents ontology terms. For example, to specify the experimental equipment (e.g., a SOLEXA sequencer) more precisely, the FuGE model uses the association "equipmentModel" between the Equipment and OntologyTerm classes to specify the equipment product name. The approach allows the flexible integration of ontological terms into the models. Both models use this mechanism to specify domain entity types or generic properties. At first glance, this is similar to the *type attributes *and generic properties presented here. However, the models do not constrain the set of allowed properties. Our approach for deriving the knowledge model from ontologies is a further development of the FuGE and MAGE-OM approach.

Another approach to utilize ontologies in domain modeling is to derive a class model from an ontology [42]. The approach defines transformation rules, which, for example, map concepts to classes and relations to associations. These rules allow the automatic conversion of ontologies into object-oriented models. However, a static class model is generated. Such a model is too inflexible to serve as a MB domain model. Changes in the ontology would require new transformations and result in new domain models. In contrast, our approach creates a dynamic representation of ontology concepts; it is a similar approach but enhances the flexibility significantly.

### Outlook and current work

#### Integration of data processing components

Another aspect of our current investigation are extension points that allow the integration of extended data processing components. These components should be seen as algorithms, formal described by type attributes. The description includes the I/O ports and configuration parameters of the algorithms, see section "Consequences for the structure of software components." This description provides enhanced capabilities in using data processing components. For example, the description of I/O ports allows an easy and semantically correct combination of processing components. Together with the formal description of exchanged data, one can ensure semantic compatibility. In this way, users will be able to join data processing components safely and create their own computing pipelines.

## Conclusion

Our aim was the development of a domain model for linear biological macromolecules that can easily integrate new or modified domain concepts and nevertheless provide a stable basis for implementation models. We achieved this through the strict separation of two model parts, the operational model and the knowledge model. These parts are connected by the meta model of the knowledge model to the whole system model.

We validated our model against the EMBL standard and by implementing an MB framework (MCK) whose data structures are based on the model. We demonstrated that our model is able to integrate new domain concepts without changes to the operational model and therefore provides a stable interface for software programs. General use of the design pattern "Dynamic Object Model" and its related patterns is the key to creating changeable, high-quality software solutions for MB.

In the future, an integration of domain-specific ontologies will enrich the model and the modeling approach presented here even further; for example, in the form of enhanced expressiveness and automatic generation of knowledge models.

The modeling approach allows the development of information systems for MB that can accommodate changes in the domain in a fast and flexible manner. This approach also provides the expressiveness and flexibility that is needed to develop a widely used domain model of MB, one which allows a shift to a global view of the domain and supports interoperability on a new level.

## Availability and requirements

Our models and the MCK are available at . MCK requires Java Software Development Kit, version 1.6 or higher, and Eclipse 3.4 .

## Authors' contributions

NB developed the modeling approach and drafted the manuscript. GW participated in developing the modeling approach and revised the manuscript. All authors read and approved the final manuscript.
